# Advances on chimeric antigen receptor-modified T-cell therapy for oncotherapy

**DOI:** 10.1186/s12943-018-0840-y

**Published:** 2018-05-16

**Authors:** Yanyu Pang, Xiaoyang Hou, Chunsheng Yang, Yanqun Liu, Guan Jiang

**Affiliations:** 1 Department of Dermatology, Affiliated Hospital of Xuzhou Medical University, Xuzhou, 221002 China; 2 Department of Dermatology, Affiliated Huai’an Hospital of Xuzhou Medical University, the Second People’s Hospital of Huai’an, Huai’an, 223002 China

**Keywords:** Chimeric antigen receptor T cells, Hematological malignancies, Acute lymphoblastic leukemia, Solid tumors, Cytokine release syndrome

## Abstract

Tumor treatment is still complicated in the field of medicine. Tumor immunotherapy has been the most interesting research field in cancer therapy. Application of chimeric antigen receptor T (CAR-T) cell therapy has recently achieved excellent clinical outcome in patients, especially those with CD19-positive hematologic malignancies. This phenomenon has induced intense interest to develop CAR-T cell therapy for cancer, especially for solid tumors. However, the performance of CAR-T cell treatment in solid tumor is not as satisfactory as that in hematologic disease. Clinical studies on some neoplasms, such as glioblastoma, ovarian cancer, and cholangiocarcinoma, have achieved desirable outcome. This review describes the history and evolution of CAR-T, generalizes the structure and preparation of CAR-T, and summarizes the latest advances on CAR-T cell therapy in different tumor types. The last section presents the current challenges and prospects of CAR-T application to provide guidance for subsequent research.

## Background

Despite the rapid development in medical science and the emergence of new medical technology, tumor therapy is still an intractable problem. Conventional therapies, such as surgery, chemotherapy, and radiotherapy, may provide short-term benefits but have annoying side effects due to their invasiveness and biotoxicity [1, 2]. Furthermore, multidrug resistance for chemotherapy and multiple toxicities of radiotherapy limit their curative effects [3, 4]. Therefore, new and effective treatments must be developed. Typical immunotherapy, including the use of tumor-infiltrating lymphocytes (TILs), T cell receptor (TCR)-engineered T cells, and chimeric antigen receptor (CAR) -modified T cells, has harnessed the immune system against cancer and emerged as a promising treatment modality for human malignancies [5–7]. TILs are cultured from fragments of resected tumors and have produced encouraging results in the therapy of metastatic melanoma [8] but are limited in other solid tumors due to the difficulty in isolation and expansion in vitro [9]. TCR T cell therapy is restricted to major histocompatibility complex (MHC)-expressing antigens [10]. Alternatively, CAR-T cell-based immunotherapy is independent of MHC [11, 12] and has achieved spectacular success in treating cancers, especially B-cell hematologic malignancies [13–15]. CARs are recombinant receptors containing an extracellular antigen recognition domain, a transmembrane domain, and a cytoplasmic signaling domain (such as CD3ζ, CD28, and 4-1BB). Therefore, T cells expressing CAR can recognize a wide range of cell surface antigens, including glycolipids, carbohydrates, and proteins [16], and can attack malignant cells expressing these antigens through the activation of cytoplasmic costimulation [12]. On July 1, 2014, the US Food and Drug Administration (FDA) granted the breakthrough therapy designation to CTL019, which is the CD19-directed CAR-T cell therapy designed by the University of Pennsylvania [17]. In 2017, FDA successively approved two drugs, namely, tisagenlecleucel (CTL019, Novartis) for the treatment of children and young adults with relapsing/refractory acute lymphoblastic leukemia (r/r ALL) and axicabtagene ciloleucel (KTE-C19, Kite Pharma) for the treatment of non-Hodgkin’s lymphomas (NHLs) [18, 19]. CAR-T cell therapy also has some effects in other diseases, such as in non-small cell lung cancer (NSCLC), malignant pleural mesothelioma (MPM), metastatic renal cell carcinoma (mRCC), and glioblastoma (GBM) [20–23]. Although the therapeutic efficacy of CAR-T cell in these solid tumors is less effective than that in hematologic diseases, the successful trials achieved by CAR-T cells provide a concrete platform for its further development in solid tumors. In this review, we analyze the reasons why CAR-T cell therapy reaches its limits when targeting solid tumors, conclude the applications of CAR-T cell therapy in different tumors, and discuss the future perspectives on CAR-T cell therapy in cancer treatment.

## CAR-T profile

### History and evolution

In 1989, as the beginning of CAR-T cell, Eshhar and colleagues first generated chimeric TCR genes that can be functionally expressed in T cells and endowed the recipient T cell with antibody-type specificity to recognize and respond to the antigen in a non-MHC-restricted manner [12]. In 1993, to achieve the advantages of antibody specificity and T-cell cytotoxic activity, Eshhar combined a single-chain variable region domain (scFv) of an antibody molecule with the constant region domain of the TCR, which is usually the ζ chain of the TCR/CD3 complex [24], to construct a chimeric receptor gene and subsequently induce the T cells to express this gene by generating chimeric scFvRζ T cells [25], which are later called “first-generation CARs” that unfortunately showed limited clinical benefit because of failure in directing T cell expansion upon repeated exposure to the antigen [26] (Table 1). Hence, a co-stimulatory signaling domain–CD28 or 41BB–was added in between scFv and CD3ζ chain to form the “second-generation CARs.” This domain sustainably activates the T cell to augment cytokine secretion and amplify T cell proliferation; thus, the T cells can expand upon repeated antigen exposure and show significant clinical responses [27, 28]. “Third-generation CARs” were formed by incorporating two or more costimulatory domains, usually CD28 and 41BB (CD137) or OX40 (CD134), into the same CAR. However, whether it has better clinical effect than second-generation CAR-T remains unclear [29]. In general, third-generation CARs enhance the expansion and persistence of CAR-T cells after tumor challenge [30, 31]. Notwithstanding, Haso et al. reported that in most vitro cases of anti-CD22 CARs for B-cell ALL, second-generation CAR was superior over third-generation CAR [32]. Moreover, a clinical trial of CEA CAR-T therapy on patients with carcino-embryonic antigen (CEA)-positive colorectal cancer (CRC) carried out by Zhang et al. showed that the third generation of CAR with CD28 and CD137 signaling does not show better performance than the second generation with CD28 signaling [33]. The significant phenotypic heterogeneity of solid tumors makes it difficult for CAR to recognize cancer cells. To circumvent these barriers in solid tumor lesions, Markus Chmielewski et al. developed the “fourth-generation CAR” (TRUCKs, T cells redirected for universal cytokine killing) that include the costimulatory domain and the CAR-inducible interleukin-12 (iIL-12) cassette. When CAR binds to target antigen, it activates T cell signaling; iIL-12 cassette then secretes pro-inflammatory IL-12, which can accumulate in the targeted tissue and thus recruit a second wave of immune cells (NK cells, macrophages) to initiate an attack toward those that would normally escape cancer cells due to the lack of CAR-recognized target and invisibility to CAR-T cells [34, 35].

**Table 1:** Summary and comparison of four generations of CAR-T therapy

CAR generations	Signal domain	Target antigen	Associated diseases	Profile	References
1st					
	CD3ζ	TAG72	Metastatic colorectal cancer	Limited persistence	[84]
	CD3ζ	FRα	Ovarian cancer	Limited persistence	[26]
	CD3ζ	L1-CAM	Metastatic neuroblastoma	Limited persistence	[85]
2nd					
	CD3ζ + CD28/CD137 (41BB)	CD19	B cell lymphomas	Enhanced expansion, persistence and anti-tumor effect	[28, 40, 86, 87]
	CD3ζ + 41BB(CD137)	IL13Rα2	GBM	Improved anti-tumor activity and T cell persistence	[22]
	CD3ζ + 41BB (CD137)	FRα	Ovarian cancer	Augmented cytokine secretion and proliferation	[88]
3rd					
	CD3ζ + CD28 + 41BB(CD137)	CD19	ALL	Superior activation and proliferation capacity	[89]
	CD3ζ + CD28 + 41BB(CD137)	PMSA	–	Promoted cytokine release, T-cell survival and tumor elimination	[90]
	CD3ζ + CD28 + CD137 (41BB)	Mesothelin	Mesothelioma	Prolonged persistence	[30]
	CD3ζ + CD28 + 41BB(CD137)	CD22	ALL	Inferior antileukemic activity	[32]
4th					
	CD3ζ + iIL-12+ co-stimulator	CEA	CEA^+^ tumors	Improved antitumor efficacy	[35]

*TAG72* tumor-associated glycoprotein 72, *CEA* carcinoembryonic antigen, *IL13Rα2* IL-13 receptor α2, *FRα* folate receptor-α, *L1-CAM* L1-cell adhesion molecule, *PSMA* prostate-specific membrane antigen

### Structure

**Fig. 1:**
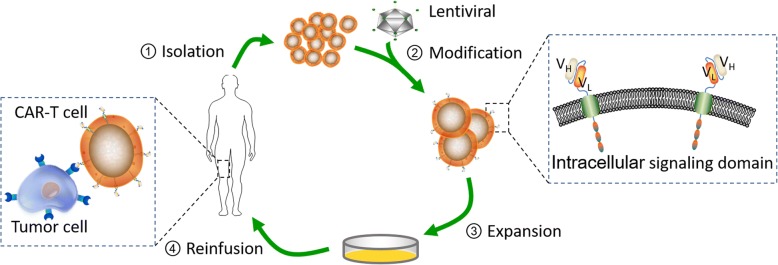
Structure and preparation of CAR-T cells. CARs can be divided into 3 main portions, that is, an extracellular antigen-binding domain followed by a space region, a transmembrane domain, and intracellular signaling domain. The four major steps are as follows: (1) isolation, in which PBMCs is harvested from the patient or donor’s peripheral blood; (2) modification, in which the T cells were activated and CARs are transduced into the activated T cells by way of lentiviral; (3) expression, in which the modified T cells expanded ex vivo to obtain clinically relevant cell numbers; and (4) reinfusion, in which the modified T cell that has reached the desired dose were reinfused into the previously lymphocyte-depleted patient

CARs are engineered receptors that possess both antigen-binding and T-cell-activating functions. Based on the location of the CAR in the membrane of the T cell, CAR can be divided into three main distinct modules (Fig. 1), that is an extracellular antigen-binding domain, followed by a space region, a transmembrane domain, and the intracellular signaling domain. The antigen-binding moiety, most commonly derived from variable regions of immunoglobulin, is composed of V_H_ and V_L_ chains that are joined up by a linker to form the so-called “scFv” [12, 25]. The segment interposing between the scFv and the transmembrane domain is a “spacer domain,” that is commonly the constant IgG1 hinge-CH2–CH3 Fc domain [36]. In some cases, the space domain and the transmembrane domain are derived from CD8 [37]. The intracellular signaling domains mediating T cell activation include a CD3ζ co-receptor signaling domain derived from C-region of the TCR α and β chains [12] and one or more costimulatory domains.

### Preparation

The manufacturing processes of CAR-T cells are complex, and we here briefly summarize their preparation. In general, the process of CAR T-cell manufacturing and delivery involves the following major steps (Fig. 1): (1) Isolation: Peripheral blood mononuclear cells are harvested from the patient or donor’s peripheral blood using a standard leukapheresis procedure, a process whereby blood is removed from an individual’s antecubital veins, separated into select components, and the remainder of the blood returned to the individual’s circulation [38]. (2) Modification: T cells were activated with CD3/CD28 magnetic beads (Dynabead) to be susceptible to viral transduction [39]. Then, CARs with the high affinity to predefined tumor antigens are transduced into these T cells by way of viral (lentiviral or retroviral) or nonviral (transposon) gene transfer systems. Lentiviral vectors and gammaretroviral vectors are currently two standard methods of viral transduction to equip T cells with a CAR [38–40]. The nonviral transduction methods usually used in engineering CAR-T cell are plasmid DNA [41] and RNA electroporation, which are also applied to T cells without pre-activation [42]. In this step, the CARs identifying tumor-associated antigens (TAAs) and, simultaneously, activating T cells were genetically expressed on the collected T cells. (3) Expansion: The CAR-T cells are expanded ex vivo to achieve the desired modified T cell dose. (4) Reinfusion: The modified T cells amplified to clinically relevant cell numbers were subsequently reinfused to the beforehand lymphocyte-depleted patient. Then, a novel CliniMACS Prodigy (Miltenyi Biotec), an automated manufacturing of CAR-T cells, has been adapted for lentiviral transduction of T cells which exhibited enormous potential [43].

## Therapeutic effect of CAR-T in different systems

Clinical trials to date have almost all focused on second- or third-generation CAR constructs. We here concluded the clinical applications of second- or third-generation CAR-T cells in different system tumors and summarized them in Table 2.

**Table 2:** Clinical trials of CAR-T therapy on different tumors

Tumors	scFv	Single domain	Dose (cells /kg or cells/ m^2^)	Clinical trials (phage and NCT number) (www.clinicaltrials.gov/)	Number of treated patients	Responses	Persistence	References
ALL	CD19	CD28 + CD3ζ	1.5 × 10^6^ to 3 × 10^6^	Phase I (NCT01044069)	5	5 CR	Uncertain^a^	[87]
ALL	CD19	CD137+ CD3ζ	1.4 × 10^6^ to 1.2 × 10^7^	Phase I (NCT01626495)	2	2 CR	One persisted 11 months, the other relapsed	[45]
ALL	CD19	41BB + CD3ζ	0.76 × 10^6^ to 20.6 × 10^6^	Phase I/ ΙΙ (NCT01626495) ( NCT01029366)	30	27 CR	2 to 3 months	[48]
ALL	CD19	CD28 + CD3ζ	3 × 10^6^	Phase I (NCT01044069)	16	14 CR	2 to 3 months	[52]
ALL	CD19	CD28 + CD3ζ	1 × 10^6^ (maximum)	Phase I (NCT01593696)	21	12 CR	Un stated	[13]
CLL	CD19	CD137+ CD3ζ	1.5 × 10^5^	Phase I (NCT01029366)	3	3CR	10 months	[46]
CLL	CD19	CD28 + CD3ζ	0.2–1.1 × 10^7^	Phase I (NCT00466531)	8	1 PR	uncertain	[91]
CLL	CD19	CD28 + CD3ζ	1 × 10^6^, 1.5 × 10^6^, 4 × 10^6^	Phase I (NCT01087294).	10	3 CR	< 1 month	[92]
CLL	CD19	41BB + CD3ζ	0.14 × 10^8^ to 11 × 10^8^	Phase I (NCT01029366)	14	4 CR, 4 PR	14 to 49 months	[49]
CLL	CD19	41BB + CD3ζ	1.6 × 10^7^; 1.0 × 10^7^; 1.46 × 10^5^	Phase I (NCT01029366)	3	2 CR, 1 PR	> 6 months	[93]
CLL/NHL/MM	κ light chain	CD28 + CD3ζ	2 × 10^7^, 1 × 10^8^, 2 × 10^8^	NCT00881920	16 (9 CLL/NHL, 7 MM)	2 CR, 1 PR	6 weeks	[94]
CLL	CD19	CD28 + 41BB+ CD3ζ	2 × 10^5^, 2 × 10^6^, or 2 × 10^7^	unstated	24	4 CR, 10 PR	6 months	[95]
MM	CD19	CD137+ CD3ζ	1 × 10^7^ to 5 × 10^7^	Phase I (NCT02135406)	10	Uncertain	–	[54]
Lymphomas	CD19	41BB+ CD3ζ	3.08 × 10^6^ to 8.87 × 10^6^	NCT02030834	28	16 CR	29.3 months	[47]
NSCLC	EGFR	CD137+ CD3ζ	0.45 to 1.09 × 10^7^	Phase I (NCT01869166)	11	2 PR, 5 SD	2 to 8 months	[20]
CCA	EGFR	CD137+ CD3ζ	2.2/2.1 × 10^6^, 1.22 × 10^6^	Phase I (NCT01869166) (NCT02541370)	1	1 PR	13 months	[63]
CRC	CEA	CD28/CD137+ CD3ζ, CD28+ D137+ CD3ζ	1 × 10^5^ to 1 × 10^8^	Phase I (NCT02349724)	10	7 SD	–	[33]
SVC	MUC1	CD28+ 4-1BB+ CD3ζ	5 × 10^5^	Phase I/II (NCT02587689)	1	Tumor necrosis	Unstated	[68]
GBM	GD2	unstated	2 × 10^7^, 5 × 10^7^, 1× 10^8^	Phase I (NCT00085930)	19	3CR	> 6 weeks	[69]
GBM	EGFRvIII	41BB + CD3ζ	1 × 10^7^	Phase I (NCT02209376)	10	1SD	–	[70]
GBM	HER2	CD28+ CD3ζ	1 × 10^6^ to 1 × 10^8^	Phase I (NCT01109095)	17	1 PR,	> 9 months	[71]
						7 SD		
GBM	IL13Ra2	41BB + CD3ζ	2 × 10^6^, 10 × 10^6^	Phase I (NCT02208362)	1	Tumor necrosis	7.5 months	[72]
Sarcoma	HER2	CD28+ CD3ζ	1 × 10^4^ to 1 × 10^8^	Phase I/II (NCT00902044)	19	4 SD	–	[74]

^a^Four of these patients were treated with subsequent HSCT

### CAR-T for hematological malignancy treatment

CAR-T cell therapy is perhaps best known for its role in the treatment of B-cell hematologic malignancies. CD19, a surface protein highly expressed on most B-lineage lymphocytes and not on normal tissue outside the B-lineage [44], is the most thoroughly studied target in all of the hematological malignancy-associated antigens, and CD19-specific CAR-T cell therapy has demonstrated enormous efficiency in inducing endurable remissions of several hematological malignancies, including ALL, chronic lymphocytic leukemia (CLL), and NHL [45–47], with complete remissions (CR) in ALL at 90% and response rates in CLL greater than 50% [48, 49]. In 2008, Till et al. reported that CD20-targeted CAR-T cells have demonstrated potential antitumor activity in treating indolent NHL and mantle cell lymphoma [50]. Later, in 2010, Kochenderfer et al. treated a patient with advanced follicular lymphoma with anti-CD19-CAR-transduced T cells, and the patient underwent a dramatic regression [51]. In 2011, Porter et al. designed a second-generation CAR-T cell in treating a patient with refractory CLL, and all three underwent CR. These findings provoked research exploring the antitumor efficacy of CD19-redirected T cells for B-cell neoplasms [46]. Based on these, in 2013, Grupp et al. extended the application of CAR-T cells to refractory B-cell ALL and established a clinical trial involving two children with ALL treated with CTL019 CAR-T cells. Surprisingly, CR was observed in both patients, demonstrating that CAR-T cells may be favorable for the treatment of patients with refractory ALL. However, cytokine-release syndrome (CRS) was also observed [45]. Subsequently, in 2014, Maude et al. conducted pilot clinical trials of 30 patients (children and adults) with r/r CD19+ ALL, in which infused autologous T cells are transduced with a CTL019. CR was achieved in 27 patients. CTL019 was effective in treating r/r ALL, even in stem cell transplantation-failed patients. Nevertheless, CRS was developed in all the patients [48]. Furthermore, Davila et al. treated 16 adult patients with r/r ALL with 19-28z CAR-T cells specific to the CD19 antigen and achieved a promising outcome, with overall CR rate of 88% (14/16). CRS, which may be related to a systemic inflammatory process induced by the reaction between infused CAR T cells and the targeted CD19 antigen, is almost inevitable. Therefore, the diagnostic criteria for severe CRS were defined, and serum C-reactive protein can serve as a reliable indicator for CRS severity [52]. Although CD19 is not an ideal antigen in multiple myeloma (MM), for its low expression in MM [53], Garfall et al. still reported that CAR-T cell therapy in conjunction with autologous transplantation has achieved durable CR in a patient with advanced MM [54]. These findings provided a road map for application of CAR-T cells in solid tumors.

### CAR-T for solid tumor treatment

The unprecedented success of CAR-T cell therapy in hematological malignancy fostered the enthusiasm to expand this technology to solid tumors. However, this therapy encountered some difficulties in application for solid malignancy. The reasons for this phenomenon are as follows: (1) lack of eligible, effective targets such as CD19 because most target antigens are more or less expressed in normal tissues; (2) hostile immunosuppressive microenvironment of solid tumors that affect the T cells; and (3) heterogeneity of solid tumors. Although the exploration of CAR-T cell treatment in solid tumors is not as definitive as in the research of hematological malignancy, some studies have achieved promising outcomes. Here, we introduced some diseases in which the CAR-T-cell treatment has exhibited benign clinical responses.

#### NSCLC

Advanced strategies, including surgery, radiotherapy, chemotherapy, and targeted therapy, have improved the survival in patients with NSCLC. Nevertheless, the 5-year survival rate of late-stage NSCLC is still unsatisfactory [55]. The breakthrough treatment of immunotherapy with CAR-T cells in hematology raised the possibility of their use in NSCLC. In 2016, Feng et al. first studied the safety and feasibility of epidermal growth factor receptor (EGFR)-targeted CAR-T cell therapy in treating 11 patients with advanced r/r NSCLC. Two patients obtained partial response (PR), and five had stable diseases (SD) after the infusion of CART-EGFR cells with mild side effects--mild skin toxicity, nausea, vomiting, dyspnea and hypotension [20]. In addition, other TAAs, like erythropoietin-producing hepatocellular carcinoma A2 (EphA2) (Li et al. 2017) [56], prostate stem cell antigen (PSCA), and mucin 1 (MUC1) (Wei et al. 2017) [57], have also been detected in NSCLC and confirmed to be promising targeting antigen for CAR-T cells. These antigen-targeted CAR-T cells have been observed to cause tumor cell lysis in vitro exerting antitumor activity in xenograft mouse models. Furthermore, targeting the combination of PSCA and MUC1 can further enhance the antitumor efficacy of CAR T cells [57].

#### MPM

MPM is an aggressive malignancy with a median survival of less than one year [58]. Mesothelin plays an important role in screening and detecting the progression of MPM [59]. Basing on this finding, some researchers considered that CAR-T targeting mesothelin can treat MPM. A phase I clinical trial conducted at the University of Pennsylvania was designed to evaluate the manufacturing feasibility and safety of mRNA-transduced CAR T cells that target mesothelin (CART-meso cells) in patients with advanced MPM. In this study, CART-meso cells showed potent antitumor activity with no distinct on target/off-tumor toxicities (pleuritis, pericarditis, or peritonitis) [60].

#### Digestive system neoplasm

Cholangiocarcinoma (CCA) is a relatively rare and aggressive malignancy of the biliary tract and is characterized by late diagnosis and poor outcomes [61]. Complete surgical resection can be used as treatment. However, most of the patients will eventually relapse because of the delayed diagnosis and advanced stage of the disease [62]. In 2017, Feng et al. applied EGFR- and CD133-specific CAR-T sequential treatments as CAR-T cocktail immunotherapy for patients with advanced unresectable/metastatic CCA. An 8.5-month PR from the initial CAR-T-EGFR treatment and another 4.5-month PR from the subsequent CD133-specific CAR-T immunotherapy were obtained. However, the epidermal and endothelial damages caused by the infusion of CAR-T cells cannot be disregarded, thereby requiring further investigation [63].

A phase I clinical trial conducted at the University of Pennsylvania was designed to evaluate the manufacturing feasibility and safety of CART-meso cells in patients with advanced MPM and explore the antitumor effect of CART-meso cells in patients with pancreatic cancer. The results showed the antitumor activity. CART-meso cells were also detected in primary and metastatic tumor sites by collecting ascites and conducting a tumor biopsy [60].

Zhang et al. established a clinical trial of CEA CAR-T therapy of 10 patients with CRC by systemic delivery through intravenous (IV) infusion to evaluate its safety and efficacy. Out of the 10 patients, 7 patients who experienced progressive disease in the previous treatments have SD after the CAR-T therapy. Moreover, severe adverse events related to CAR-T therapy are not observed [33].

#### Genitourinary system diseases

Epithelial ovarian cancer (EOC) remains to be the most mortal of all gynecological malignancies mainly due to its subtle nature. Despite the fact that most patients with EOC yield a good clinical response following current advanced therapy, almost all patients will ultimately relapse and eventually develop drug resistance [64]. The survival of patients with EOC is positively related to the presence of TILs, which play a significant role in adoptive T-cell therapy [65]. MUC16, a well-known ovarian tumor antigen, is overexpressed by a majority of EOC but at a low level on normal tissues [66]. On the basis of this rationale, Brentjens et al. developed T cells expressing MUC16 to treat EOC. Moreover, to overcome the hostile tumor environment, they co-expressed IL-12 on T cells. Hence, a clinical trial testing the safety of IV and intraperitoneal infusion of genetically modified autologous T cells expressing MUC16 and secreting IL-12 in patients with EOC was conducted. The result demonstrated that the intraperitoneal injections of CAR T cells are superior to that of IV alone [67].

You et al. launched a phase I clinical trial to evaluate the ability of engineered CAR-T cells targeting MUC1 to treat patients with seminal vesicle cancer (SVC). To suppress the unfavorable tumor microenvironment, they induced IL-12 co-expression and constructed two anti-MUC1 CAR-T cell lines, that is, SM3-CAR (co-expressing IL-12) and pSM3-CAR (without IL-12). These two types of CAR-T cells were injected intratumorally into two separate metastatic lesions of the same patient with MUC1^+^ SVC as part of an interventional treatment strategy. The results showed tumor necrosis induced by pSM3-CAR is more evident than that by SM3-CAR, without significant side effects [68].

#### GBM

CAR-T cell has also been explored in recent years to treat central nervous system cancers. In 2011, Louis et al. conducted a clinical trial of GD2-specific CAR-T therapy in 19 patients with high-risk neuroblastoma. Three patients had a CR to CAR-T cell infusion, with only slight fever and light-to-moderate local pain being observed [69]. In 2017, a clinical trial of IV administration of EGFRvIII-specific CAR-T cells for the treatment of 10 patients with refractory GBM was established at the University of Pennsylvania. The infusion of CAR-T cells was feasible and safe, without evident off-tumor toxicity or CRS [70]. Furthermore, an open-labeled phase 1 dose-escalation study was conducted at the Baylor College of Medicine, Houston Methodist Hospital, and Texas Children’s Hospital to evaluate the safety and anti-GBM activity of HER2-specific CAR-modified virus-specific T cells in patients with progressive GBM. The results showed that the infusions are well tolerated, with no dose-limiting toxic effects. Moreover, 1 patient showed a PR for more than 9 months, whereas 7 patients had SD for 8 weeks to 29 months [71]. In addition, Brown et al. initiated a clinical trial with one patient with relapsing GBM, who received administration of IL13Rα2 targeted IL13BBζ–CAR-T cells, and regression of tumors was observed and persisted for 7.5 months after the administration of CAR-T cell therapy [72].

#### Sarcomas

Sarcoma, which can be located anywhere in the body, is usually treated with surgical resection, with or without radiotherapy, and chemotherapy. However, patients with advanced stage sarcomas still have poor prognosis [73]. Hence, several researchers speculated that CAR-T-cell treatment may benefit patients with sarcoma. In 2015, Ahmed et al. designed a phase I/ II clinical study to evaluate the safety and efficacy of HER2-specific CAR-T cells in patients with r/r HER2-positive sarcoma. A total of 19 patients were enrolled in this research, and they received escalating doses of HER2-specific CAR-T cells. Although no CR were observed, 4 out of 17 patients that can be evaluated have SD for 12 weeks to 14 months [74].

## Conclusion

In this review, we summarized the current clinical studies on CAR-T treatment of hematologic diseases and solid tumors. Clinical outcomes of CAR-T cell therapy in patients with hematologic malignancies have been encouraging. However, in patients with solid tumors, the outcomes have been discouraging, nevertheless, not gloomy. CAR-T cell therapy, as a promising treatment, has the following advantages: (1) binding surface antigen of tumors in non-MHC restriction manner; (2) recognizing multiple antigens simultaneously; and (3) obtaining a large number of CAR-T cells ex vivo in a short term. CRS is an ineluctable complication of CAR-T-cell therapy on the basis of the clinical trials of hematological malignancies mentioned above. The manifestations of CRS include fevers, hypotension, nausea, myalgias, and neurologic dysfunction. When CRS is severe, vasopressors, mechanical ventilation, antiepileptics, and hemodialysis may be required [52]. Fortunately, researchers can now control most cases of CRS with an anti-interleukin 6 antibody, such as tocilizumab, which was approved by the FDA for the treatment of CAR-T-cell therapy-induced CRS in August 2017 [75]. The CRS is not common in solid tumors treated by CAR-T cells; however, on target/off-tumor toxicity has become common due to unavoidable expression, to some extent, of target antigens in normal tissues [60]. This phenomenon could be solved by manufacturing CAR-T cells with dual antigen specificity or switchable dual-receptor [76, 77] or by transfecting T cells with mRNA encoding CAR to reduce their half-life; these CAR-T cells can be repeated administered, and the toxicity to normal tissues can be mitigated [78]. Moreover, the hostile immunosuppressive microenvironment is one of the major challenges in CAR-T-cell treatment of solid tumors. The tumor microenvironment is a complex and dense fibrotic matrix network composed of malignant and nonmalignant cells, in which the infiltrated CAR-T cells can be inhibited by immunosuppressive cells such as regulatory T cells (Tregs) and myeloid-derived suppressive cells [79]. In a recent study conducted by Chen et al., CARs were engineered to target a bunch of soluble ligands, including TGF-β, an otherwise immunosuppressive factor in a variety of solid tumors, and demonstrated the ability to effectively convert TGF-β from a potent immunosuppressive cytokine to a strong stimulant for the primary human T cells [80]. Another research by Batchu et al. also shed light on the immunosuppressive microenvironment in solid tumors. They discovered that suppressing interleukin-10, an immune inhibitory cytokine secreted by Tregs and pancreatic cancer cells, can reverse the negative effect of the tumor microenvironment on mesothelin-CAR-T cells in pancreatic cancer in vitro [81]. In addition, solid tumors have relatively limited body distribution and are concrete compared with hematological malignancies. Hence, we can hypothesize that in some cases, the regional delivery of CAR-T cells may be immensely superior to systemic administration. Several studies reported that local injection, such as intrapleural administration and local intracranial delivery, show greater potential than IV injection [23, 82]. These findings indicated that CAR-T-cell therapy can gain momentum to break through the restriction of tumor microenvironment in treating solid malignancies. In the aggregates, CAR-T-cell therapy is a promising strategy against neoplasms. Several key points must be considered to translate the success of CAR-T cell therapy to extensive solid tumors. These widely acknowledged key points include finding a specific antigen or engineering multiple antigen-targeted CAR-T cells, directly targeting the constituent of immunosuppressive microenvironment, and creating a suitable tumor microenvironment [83]. In addition to these factors, replacing IV infusion with regional delivery, transfecting T cells with mRNA encoding CAR, and combining T cells with oncolytic viruses or immune-checkpoint blockade to bolster the potency of CAR-T cells can also be considered.

## Abbreviations

ALL: Acute lymphoblastic leukemia
CAR: Chimeric antigen receptor
CCA: Cholangiocarcinoma
CEA: Carcinoembryonic antigen
CR: Complete remissions
CRC: Colorectal cancer
CRS: Cytokine-release syndrome
EGFR: Epidermal growth factor receptor
EOC: Epithelial ovarian cancer
EphA2: Erythropoietin-producing hepatocellular carcinoma A2
FDA: Food and Drug Administration
FRα: Folate receptor-α
GBM: Glioblastoma
IL13Rα2: IL-13 receptor α2
L1-CAM: L1-cell adhesion molecule
MHC: Major histocompatibility complex
MM: Multiple myeloma
MPM: Malignant pleural mesothelioma
mRCC: Metastatic renal cell carcinoma
MUC1: Mucin 1
NHLs: non-Hodgkin’s lymphomas
NSCLC: Non-small cell lung cancer
PR: Partial response
PSMA: Prostate-specific membrane antigen
scFv: Single-chain variable region domain
SR: Stable diseases
SVC: Seminal vesicle cancer
TAAs: Tumor-associated antigen
TAG72: Tumor-associated glycoprotein 72
TCR: T cell receptor
TILs: Tumor-infiltrating lymphocytes
